# International Medical Congress

**Published:** 1881-10

**Authors:** 


### EDITORIAL DEPARTMENT.

"If thou hast truth to utter, speak:
And leave the rest to God."

THE INTERNATIONAL MEDICAL CONGRESS.—The seventh meeting of the International Medical Congress was held in London, August 3-9. It was a gathering that will be memorable in the history of medical progress. From all parts of the civilized globe the representative men of the profession, the leaders in medical thought, came to the great conference in the intellectual centre of the world.

It is yet too early to speculate upon the results that will probably flow from the interchange of thought, observation and experience that took place. One thing seems certain; notwithstanding the strong defence of Volkmann, the elaborate ceremonial of Listerism appears to have received its quietus in the discussion in which Keith, Spencer Wells, and Lister himself took part. By checking the statements of

Volkmann with those of Keith in this discussion, the impartial reader will be strongly tempted to conclude that the improvement in results obtained since the introduction of the antiseptic system in surgery is due far more to cleanliness than to any bactericide property of carbolic acid, thymol, chloride of zinc, oil of eucalyptus, or whatever antiseptic may be used.

On the other hand, the truth of the germ theory upon which the antiseptic system was so logically based, has received extraordinary support from the researches of Pasteur on the prophylactic value of vaccination, with a modified virus, against chicken cholera and splenic fever, and the demonstrations of Dr. Koch, of Berlin, on the nature and development of pathogenic bacteria. Thus, while the creed upon which the ceremonial is based has been almost objectively established, the ceremonial itself has received a sad set-back. We may expect to hear "*Fort mit dem Spray!*" from many clinical and operating rooms this winter.

The addresses in the general meetings were all excellent in matter and presentation. The presidential address of Sir James Paget was a happy introductory to those that followed. That of Dr. Billings, of our National Medical Library, on Medical Literature, was a characteristic example of the author's mastery of all the details of medical bibliography. His four rules for the preparation of an article for a medical journal, although they have already been quoted in nearly every journal published, will bear repetition. They are as follows:

"First, have something to say.
Second, say it.
Third, stop as soon as you have said it.
Fourth, give the paper a proper title."

Medical literature would be of far more value, and certainly much less voluminous, if these four simple rules were always observed.

Virchow made a brave and stirring plea for the freedom of scientific inquiry. His address was on "The Value of Pathological Experiment," and secured, as it deserved, the hearty endorsement of the Congress.

But, as at all well conducted feasts, the best things are reserved for the last, so also in the Congress the best address was probably that delivered on the last day of the meeting by Prof. Huxley, on "The Relations of Biological Science to Medicine." In his clear, logical, definite way, which is so nearly allied to true eloquence, nay, which is perhaps the truest eloquence, the English "Altmeister" of biology pointed out the relations between anatomy, physiology and pathology in the broadest sense of the terms. At the close of his address he indulged in some prophesies, which, while enthusiastically applauded, must have caused a doubt of their fulfillment to arise in many of his listeners.

One of the sections (Sect. XII.) was devoted to the consideration of the Diseases of the Teeth. It was gracefully presided over by Mr. Edwin Saunders, F. R. C. S., who delivered an excellent address upon the position of Dental Surgery in Great Britain.

To make mention even of the work done in the various sections would carry us far beyond our limits.

The United States were represented at the Congress by some of the best men in the profession, who, with one conspicuous exception, reflected credit upon the American name abroad.

